# Assessment of sensitivity to common aeroallergens in a Tunisian population

**DOI:** 10.1186/1939-4551-8-S1-A255

**Published:** 2015-04-08

**Authors:** Saleh Alwasel

**Affiliations:** 1 King Saud University, Saudi Arabia

## 

Allergic diseases are common in clinical practice and are considered by the World Health Organization (WHO) as an important health problem (Liu et al. 2010).Worldwide, about 500 million people are affected (15%) with a prevalence of 20-30% in developed countries (Laaidi et al. 2009). This study aimed to identify the major aeroallergens and their prevalence in a population of patients living in a polluted oasis situated in the southwest of Tunisia. A total of 67 patients were admitted to the Department of Pneumology and Oto-rhino-laryngology at Gafsa Hospital between August 2007 and September 2008. They were invited to a health examination including skin-prick test, blood sampling and assessment of specific IgE to several common aeroallergens. Of the 67 patients, only 39 were sensitive to allergens (58.20%), 23 of whom were sensitive to more than one type of allergen (58.97%). The most common aeroallergens were tree and grass pollen (32.96%), followed by animal dander (19.78%), mites (17.58%), herbaceous pollen (12.08%), mould (6.59%), latex (6.59%) and cockroaches (4.39%).

Although this study was limited to a modest target population of patients, we did observe significant results that highlight the most frequently detected allergens in the region of Gafsa. These results are in agreement with reports from other studies conducted in other areas.
